# Draft Genome Sequence of *Lactococcus garvieae* Strain PAQ102015-99, an Outbreak Strain Isolated from a Commercial Trout Farm in the Northwestern United States

**DOI:** 10.1128/genomeA.00781-16

**Published:** 2016-08-04

**Authors:** Michael C. Nelson, Jed S. Varney, Timothy J. Welch, Joerg Graf

**Affiliations:** a Department of Molecular and Cell Biology, University of Connecticut, Storrs, Connecticut, USA; b Pacific Aquaculture, Nespelem, Washington, USA; c National Center for Cool and Cold Water Aquaculture, Agricultural Research Service, U.S. Department of Agriculture, Kearneysville, West Virginia, USA

## Abstract

We announce the draft genome assembly of *Lactococcus garvieae* strain PAQ102015-99, a recently isolated strain from an outbreak of lactococcosis at a commercial trout farm in the northwestern United States. The draft genome comprises 14 contigs totaling 2,068,357 bp with an *N*_50_ of 496,618 bp and average G+C content of 38%.

## GENOME ANNOUNCEMENT

*Lactococcus garvieae*, the causative agent of lactococcosis, is a highly virulent Gram-positive pathogen of rainbow trout (*Oncorhynchus mykiss*) and is recognized as one of the most significant disease threats to the European trout farming industry (1, 2). Lactococcosis was originally identified in Italy and Spain but rapidly spread throughout most of Europe and the United Kingdom and subsequently has been documented in Australia and South Africa (3–5). *L. garvieae* has also been a major cause of loss in cultured marine fish in Asia (6) and strains of *L. garvieae* have been associated with disease in other animals, humans, and isolated from human food sources (7, 8). Recently, we identified an outbreak of lactococcosis at a rainbow trout farm in the Pacific Northwest region of the United States. Like European strains of *L. garvieae*, outbreak strains in the United States are highly virulent for rainbow trout and cause high mortality in infected stocks.

Here, we report the draft genome sequence of a representative United States *L. garvieae* isolate (strain PAQ102015-99), which was isolated from the head kidney of a rainbow trout showing clinical signs of lactococcosis. Whole-genome shotgun sequencing was conducted using an Illumina MiSeq on a NexteraXT prepared library. Quality filtering, trimming, and *de novo* assembly were performed using CLC Genomics Workbench v8.5.1. Functional annotation was conducted with Prokka (9), using currently available *L. garvieae* genomes as the initial reference database for annotation.

The draft genome consists of 14 contigs totaling 2,068,357 bp with an *N*_50_ value of 496,618 and average G+C content of 38.0%. A total of 1,947 predicted CDSs, 47 tRNAs, and 5 rRNAs were identified after annotation. Six hundred fourteen CDS features were identified as hypothetical proteins while 31 were identified as phage related. Important features related to pathogenicity and virulence included genes for colicins and related iron acquisition and metabolism, antibiotic resistance, cell adherence and invasion, and hyaluronic acid metabolism. Availability of this United States strain genome sequence will allow comparison to the genomes of European and Asia strains and thus provide a better understanding of the evolutionary relationship among these strains and insight into the emergence of this pathogen in the United States.

### Accession number(s).

The raw sequencing and contig data are available from GenBank/ENA/DDBJ under BioProject accession number PRJNA320828. The annotated assembly is directly available under accession number LXWL00000000.
